# Some Suggestions on Metal Cap Crowns

**Published:** 1889-02

**Authors:** W. Mitchell


ARTICLE VI.

### SOME SUGGESTIONS ON METAL CAP CROWNS.

BY MR. W. MITCHELL.

He did not propose to go into the evolution of crown work, as that was already too well known to the educated dentist to require even a passing notice.

He considered that no method of crowning teeth or roots gave more satisfactory results than that of the metal cap crown, owing to its cleanliness, its indestructibility, and its adaptability to the greatest variety of cases. Its capacity for restoring articulating surfaces was limited only by the skill of the dentist. The object of his paper was to discuss the *practical* part of the subject which hitherto, he thought, had been somewhat neglected.

If the tooth or root were in a healthy condition, he proceeded to prepare it for the cap. This might be done in various ways, principally by means of corundum wheels used on the engine, for the purpose of restoring the bite or bringing about adjustment between adjacent teeth. In bicuspids or molars, when the root was on a line with the gum, he found it a good plan to put one, two, and even three good platina or dental alloy pins in the pulp canals, first fitting them and making them the proper length, and then driving them home; this gave support to the cap, especially when it had much work to do. Having coned the root or tooth, he carefully removed all enamel remaining round the cervical margin with an instrument, by inserting the point of it under the free margin of the gum, and with a draw-cut rapidly trimming off the enamel. The most careful preparation of the neck of the tooth was essential to the success of the operation, for the fit and ultimate retention of the cap depended upon it. The tooth was then ready for the cap, for which he used coin gold, rolled to No. 5 B P G for the band, and No. 3 for the top; he preferred this because of its uniformity in working, its colour in the mouth, and its durability. In preparing the band he used no model other than the tooth itself, and contended that this was the only way to get a perfect fit; another reason being that it could then be more easily determined how to trim the band to adapt it to the requirements of the gum. He next took a piece of visiting card and approximated it to the height and length for the plate required for the band; then, with a pair of moderate-sized pliers, having one jaw flat and the other half round, he proceeded to adapt the plate to the tooth, making the plate slightly flaring towards the grinding surface. When he had fitted the crown as well as possible, he closed it a little by squeezing between the fingers, so that it sprang over the tooth when put on for trial; should it not pass evenly under the gum, it should be trimmed until it followed the contour of the gum.

He recommended manicure scissors with short but heavy blades as the most suitable for cutting the gold. A line should be left for lapping, and the lap should be left on the outside for convenience in marking and trimming. Having marked along the lap with the point of the instrument, the band should be removed from the tooth, closed about a line, and soldered, which was most easily done over an ordinary Bunsen. He preferred a lap joint to an edge one, because it was more easily made, and saved time. The band might now be tried on; if too tight, it should be stretched to the required size by lightly hammering it on the horn of an anvil. He then chamfered the edge of the band that went under the gum and burnished it. After the band was started he assisted it to where it was ultimately wanted by driving it on with a steel mallet. Having judged the height the band should be left, he trimmed if necessary, and contoured, after which, he flattened the surface of the band by holding its top edge against the flat side of a corundum wheel and giving it a slight rotary motion.

The plan he recommended as the simplest for making the tops was to take the teeth that had been extracted, or models for impressions of natural teeth, invest the roots and crowns in plaster almost to the grinding surface, leave the block about two inches high, and trim, leaving a base about an inch square. He found squaring and chamfering the corners left it the best shape for drawing for the marble dust mould. The face of the block should be made flat, and left 1-16 in. wide. After securing the model, a sand—or, better—marble dust mould was made; this was finished with file and graver, and polished on a lathe.

No counter die was needed for producing the tops. Simply place the gold to be stamped on a piece of lead, a smooth surface could be easily obtained by a blow with a hammer. He recommended putting a heavy piece of tin-foil between the gold and the lead, which prevented any small particle of lead adhering to the gold and producing sweating during the subsequent stages of soldering. After swaging the top, the narrow flat margin might be utilized to hold it during soldering. Flush the top with 20-carat solder over a bench Bunsen. Having done this, touch the flat edge of the band with borax, place in the desired position on the flushed side of the top, return to the flame, when the top and band would soon unite most satisfactorily; drop into the pickle while hot to remove borax. Trim off surplus with plate shears, then grind off overhanging edges to the required contour, polish same as a gold plate minus stoning down, and the cap would be ready for final adaptation. From experience it would be found that no special vent for surplus cement would be needed. He recommended Richmond's crown and Welch's crown cements as the best. It should be mixed so that it would drop from the spatula. In conclusion, he claimed that his method was the simplest, easiest and most expeditious.—*Dental Record.*

